# Cimetidine and Clobenpropit Attenuate Inflammation-Associated Colorectal Carcinogenesis in Male ICR Mice

**DOI:** 10.3390/cancers8020025

**Published:** 2016-02-20

**Authors:** Takuji Tanaka, Takahiro Kochi, Yohei Shirakami, Takayuki Mori, Ayumi Kurata, Naoki Watanabe, Hisataka Moriwaki, Masahito Shimizu

**Affiliations:** 1 Department of Diagnostic Pathology (DDP) and Research Center of Diagnostic Pathology (RC-DiP), Gifu Municipal Hospital, 7-1 Kashima-cho, Gifu City, Gifu 500-8513, Japan; mulmirry@yahoo.co.jp (A.K.); naoki@watanabe.name (N.W.); 2 Department of Tumor Pathology, Gifu University Graduate School of Medicine, 1-1 Yanagido, Gifu City, Gifu 501-1194, Japan; 3 Department of Gastroenterology/Internal Medicine, Gifu University Graduate School of Medicine, 1-1 Yanagido, Gifu City, Gifu 501-1194, Japan; kottii924@yahoo.co.jp (T.K.); shirakamiyy@yahoo.co.jp (Y.S.); hmori@gifu-u.ac.jp (H.M.); shimim-gif@umin.ac.jp (M.S.); 4 Department of Pharmacy, Ogaki Municipal Hospital, 4-86 Minaminokawa-cho, Ogaki 503-8502, Japan; ta_mori04@yahoo.co.jp

**Keywords:** histamine receptors, antagonists, chemoprevention, colorectal cancer, azoxymethane, dextran sodium sulfate, inflammatory bowel disease

## Abstract

Histamine and histamine receptors (Hrhs) have been identified as critical molecules during inflammation and carcinogenesis. This study was conducted to determine the effects of Hrh1-Hrh3 antagonists on inflammation-associated colorectal carcinogenesis. Male ICR mice were treated with azoxymethane (AOM, 10 mg/kg bw, i.p.) and 1.5% dextran sodium sulfate (DSS, drinking water for 7 days) to induce colorectal carcinogenesis. The mice were then fed diets containing test chemical (500 ppm terfenadine, 500 ppm cimetidine or 10 ppm clobenpropit) for 15 weeks. At week 18, feeding with the diets containing cimetidine (Hrh2 antagonist) and clobenpropit (Hrh3 antagonist/inverse agonist) significantly lowered the multiplicity of colonic adenocarcinoma. Terfenadine (Hrh1 antagonist) did not affect AOM-DSS-induced colorectal carcinogenesis. Adenocarcinoma cells immunohistochemically expressed Hrh1, Hrh2, Hrh3 and Hrh4 with varied intensities. Because clobenpropit is also known to be a Hrh4 receptor agonist, Hrh2, Hrh3 and Hrh4 may be involved in inflammation-related colorectal carcinogenesis. Additional data, including the mRNA expression of pro-inflammatory cytokines and inducible inflammatory enzymes in the colonic mucosa, are also presented.

## 1. Introduction

A strong association between inflammation and cancer in several tissues has been suggested [1,2]. Patients with inflammatory bowel disease (IBD), especially ulcerative colitis (UC) and Crohn’s disease (CD), have a significantly increased risk of developing premalignant lesions (dysplasia) and malignancy (adenocarcinoma, ADC) in the colorectum [3]. Although UC-associated colorectal cancer (CRC) accounts for less than 2% of all CRCs in the general population, UC-associated CRC is responsible for 10%–15% of deaths in UC patients [4]. Increases in the degrees of inflammation and the disease duration could increase the risk of CRC in UC patients [5]. CD patients also have an increased risk of large and small bowel ADC [6]. Like in Western countries, the number of UC patients as well as CRC patients have continued to increase in Asian countries, including Japan [7]. Therefore, it is necessary to investigate the precise mechanisms of CRC development in the inflamed colon to establish preventive strategies, such as chemoprevention [8]. For this purpose, we have developed a novel animal model, azoxymethane (AOM)/dextran sodium sulfate (DSS) murine model of colitis-related colorectal carcinogenesis [9]. Using this model [9], we previously demonstrated that the chemopreventive efficacy of ursodeoxycholic acid (UDCA) is superior to that of 5-ASA [10].

In the inflammatory microenvironment, infiltrated macrophages and mast cells produce inflammatory mediators to promote tumor growth in tissues. Histamine, the first discovered mediator in mast cells, plays an important role as a neurotransmitter and chemical mediator in multiple physiological and pathophysiological processes in tissues [11]. Following the identification of four histamine receptors (Hrh1, Hrh2, Hrh3 and Hrh4) using selective agonists and antagonists [12], the roles of histamine and histamine receptors have been reported in IBD [13]. In our previous study using the AOM/DSS model, mice lacking mast cells were less susceptible to inflammation-associated colorectal carcinogenesis [14]. Additionally, mast cells and their specific cytokines could play an important role in inflammation-mediated tumorigenesis through the regulation of pro-inflammatory cytokines and inducible inflammatory enzymes, such as cyclooxygenase (COX)-2 and inducible nitric oxide synthase (iNOS). In this AOM/DSS model, oxidative/nitrosative DNA damage occurred in the inflamed colon due to DSS exposure [15]. Both inducible inflammatory enzymes (COX-2 and iNOS) and pro-inflammatory cytokines (tumor necrosis factor (TNF)-α, interleukin (IL)-1β and IL-6) play a key role in the pathogenesis of intestinal damage in IBD [16] and IBD-associated CRC [17]. Therefore, controlling inflammation in the colorectum may lead to the prevention of inflammation-associated CRC [18].

In the gastrointestinal tract, histamine is synthesized by histidine decarboxylase (HDC) and stored in various cell types, including mast cells [19]. Elevated concentrations of histamine have been shown in various inflammatory and neoplastic diseases, such as UC, CD and CRC [20]. The stimulatory effect of histamine on tumor growth is also well recognized [21]. The levels of HDC mRNA protein and its enzymatic activity are significantly increased in both experimental and human cancers, including CRC [20,22]. The biological function of histamine is mediated through at least four different histamine receptors, Hrh1-4, which are members of the G-protein-coupled receptor (GPCR) family [19,22,23,24,25,26,27]. Hrh4 also plays a role in proliferation of both normal and malignant cells [19]. Exogenous histamine is able to stimulate CRC implant growth [27]. Boer et al. previously reported the immunohistochemical expression of Hrh1, 2 and 4 in CRC and the adjacent normal mucosa [24]. Their findings suggested that mast cells, histamine and/or Hrh are involved in carcinogenesis in the colorectum with or without chronic inflammation. Recently, an interesting study demonstrated that HDC-knockout mice that received AOM and DSS have a high rate of CRC [28].

In the current study, we aimed to determine whether antagonists of Hrh1 (terfenadine [29,30,31]), Hrh2 (cimetidine [23,26]) and Hrh3 (clobenpropit) affect colorectal carcinogenesis using a murine model of inflammation-associated colorectal carcinogenesis [9,15] in Experiment 1 (the 18-week experiment), since an association between histamine receptors and cancer is suspected [21]. In Experiment 2 (the 4-week experiment), the effects of dietary terfenadine, cimetidine and clobenpropit on the mRNA expression of inducible inflammatory enzymes (COX-2 and iNOS) and pro-inflammatory cytokines (TNF-α, IL-1β and IL-6) in the colorectum of mice were assessed. Clobenpropit is known to be a specific Hrh3 antagosnist/inverse agonist and a partial agonist at Hrh4. An abnormal expression of Hrh4 was reported in CRC [32] and treatment with clobenpropit was able to suppress several types of cancer development [33,34]. The effects of these antagonists on the expression of COX-2, iNOS, TNF-α, IL-1β and IL-6 in the colonic mucosa were also assayed in a 4-week short-term experiment. We also investigated the effects of three antagonists of Hrh1, Hrh2 and Hrh3 on the derivatives of reactive oxygen metabolites (d-ROMs) [35], as an index of products of reactive oxygen species (ROS), and biological antioxidant potential (BAP) [36], as a biomarker of antioxidant potential. The d-ROM test utilizes *N, N*-diethyl-*para*-phenylendiamine (DEPPD), which reacts with free radicals to form a colored radical detectable at 505 nm as a result of a linear kinetic reaction. The BAP test was used to measure the antioxidant capacity in the body by measuring the ratio of ferric ion (Fe^3+^) to ferrous ion (Fe^2+^).

## 2. Materials and Methods

### 2.1. Animals, Chemicals and Diet

Male Crj:CD-1 (ICR) mice (Charles River Japan, Inc., Tokyo, Japan) were used in this study. All mice were maintained in the animal facility of the Division of Animal Experiment, Life Science Research Center, Gifu University, according to the Institutional Animal Care Guidelines. Animals were housed in plastic cages (four mice/cage) with *ad libitum* access to tap water and a basal diet (CRF-1, Oriental Yeast Co., Ltd., Tokyo, Japan), under controlled conditions of humidity (50% ± 10%), light (12/12 h high/dark cycle) and temperature (23 ± 2 °C). All animals were quarantined for the first seven days just after arrival and randomized by body weights into the experimental groups. AOM and DSS (with a molecular weight of 36,000–50,000) were purchased from Sigma-Aldrich Chemical Co. (Cat. No. A5486; St. Louis, MO, USA) and MP Biomedicals, LLC (Cat. no. 160110; Aurora, OH, USA), respectively. DSS was dissolved in distilled water at a concentration of 1.5% (w/v) just before use. All animal experiments and study designs were approved by the Experimental Animal Research Committee of Gifu University. All animal handling and procedures were performed in accordance with the appropriate Institutional Animal Care Guidelines.

### 2.2. Experiment 1 (the Eighteen-Week Experiment)

A total of 112 male ICR mice, five weeks of age, were used in this study. They were divided into eight experimental and control groups: group 1, AOM/1.5% DSS (n = 20); group 2, AOM/1.5% DSS/500 ppm terfenadine (n = 20); group 3, AOM/1.5% DSS/500 ppm cimetidine (n = 20); group 4. AOM/1.5% DSS/10 ppm clobenpropit (n = 20); group 5, 500 ppm terfenadine alone (n = 8); group 6, 500 ppm cimetidine alone (n = 8); group 7, 10 ppm clobenpropit alone (n = 8), and group 8, untreated (n = 8). The mice were initially treated with a single subcutaneous (s.c.) injection of AOM (10 mg/kg bw) followed by 1.5% DSS in their drinking water for seven days to induce colorectal tumors. They were then given a basal diet containing 500 terfenadine, 500 cimetidine or 10 ppm clobenpropit for 15 weeks. Basal diet CRF-1 was used during the study. All mice were sacrificed at week 18 by an overdose of ether for pathological examinations. At the time of sacrifice, a complete necropsy was performed on all animals. The weights of the whole body, liver, spleen, and kidneys and the length of the large bowel were measured. All tissues were then fixed in 10% buffered formalin for over 24 h. After careful macroscopic observation, tissues from the large bowel, liver, spleen, and kidneys were processed for histopathological examinations on paraffin-embedded sections (3 μm in thickness) after hematoxylin and eosin (H&E) staining by a certified pathologist (T.T.).

#### 2.2.1. Counting the Number of Mucosal Ulcers in the Colorectum

After macroscopic observation for ulcers in the large bowel fixed in 10% formalin, the number of mucosal ulcers was carefully counted on H&E-stained sections from all groups. The measurements were performed on at least five sections from the entire colorectum with or without proliferative lesions and were expressed as a mean number/mouse.

#### 2.2.2. Immunohistochemical Analysis of Minichromosome Maintenance Protein 2 (MCM2), Cleaved Caspase-3, Hrh1, Hrh2, Hrh3 and Hrh4 in Colonic Adenocarcinoma

Paraffin-embedded sections from the colorectum containing tumors from the mice in groups 1 through 4 were used for an immunohistochemical investigation. Serial histological sections (4 μm thick) were made for immunohistochemistry. Immunohistochemical staining was performed using an automated system (Ventana BenchMark XT system; Ventana, Touchstone, AZ, USA), according to the manufacturer’s instruction. The primary antibodies used included an anti-MCM2 rabbit monoclonal antibody (no. 3619, anti-MCM2 (D7611)XP, 1:400 dilution; Cell Signaling Technology, Inc., Danvers, MA, USA), anti-cleaved caspase-3 rabbit polyclonal antibody (#9661, 1:200 dilution; Cell Signaling Technology), anti-Hrh1 goat polyclonal antibody (PAB6855, 1:250 dilution; Abnova, Taipei City, Taiwan), anti-Hrh2 rabbit polyclonal antibody (NBP1-86082, 1:50 dilution; Novus Biologicals, LLC, Littleton, CO, USA), anti-Hrh3 rabbit polyclonal antibody (PAB3659, 1:25 dilution; Abnova) and anti-Hrh4 rabbit polyclonal antibody (HPA035009, 1:200 dilution; ATLAS Antibodies AB, Stockholm, Sweden). For each case, the positive and negative controls were concurrently stained. Mouse brain was used for positive controls. Finally, the sections were lightly counterstained with Mayer’s hematoxylin (Merck, Tokyo, Japan). Immunoreactivity for the antibodies against MCM2 and cleaved caspase-3 was assessed in colonic adenocarcinomas (>3 mm in diameter) that developed in groups 1 (n = 11), 2 (n = 13), 3 (n = 5) and 4 (n = 6), using a microscope (Olympus BX41, Olympus Optical Co., Tokyo, Japan). The intensity and localization of immunoreactivity against the primary antibodies were determined by a pathologist (T.T.) who was blinded to the treatment groups. The number of nuclei with positive reactivity for MCM2 and cleaved caspase-3 was counted in a total of 3 × 100 cells in three different areas of colonic cancer and was expressed as a percentage (mean ± SD).

#### 2.2.3. Oxidative Stress and Antioxidant Status Measurements

Blood samples were taken from all mice at sacrifice after overnight fasting (18-week and 4-week experiments). Both the oxidative stress level and antioxidant properties in the serum were determined by an automated method as described previously. In brief, the oxidative stress level was assessed by a d-ROM test (Diacron s.r.l. Grosetto, Italy) [35] and the antioxidant status was determined using a BAP test (Diacron s.r.l.) [36]. Measurements obtained in the d-ROM and BAP tests were expressed as Carratelli units (Carr U), with 1 Carr U equal to 0.08 mg/100 mL H_2_O_2_ [37] and μmol/L, respectively. The d-ROM level represents the total level of peroxidized metabolites. Both tests were performed with an automated clinical chemistry analyzer [36]. The d-ROM/BAP ratio, which was calculated using the values obtained in the d-ROM and BAP tests, indicates the degree of latent antioxidant potential.

### 2.3. Experiment 2 (the Four-Week Experiment)

A total of 40 male ICR mice, 5 weeks of age, were used. They were divided into five groups (8 mice each): group 1, 1.5% DSS in drinking water for 7 days; group 2, 1.5% DSS and 500 ppm terfenadine; group 3, 1.5% DSS and 500 ppm cimetidine; group 4, 10 ppm of clobenpropit; and group 5, untreated control. The experimental diet containing terfenadine, cimetidine or clobenpropit was mixed with a basal diet, CRF-1, and given to the mice during the study (for 4 weeks). The mRNA expression of COX-2, iNOS, TNF-α, IL-1β and IL-6 [38] in the colorectal mucosa was assayed at the end of the study (week 4).

### 2.4. Total RNA Extraction and Quantitative Real-Time (RT)-PCR

Total RNA was extracted from the colorectal mucosa using the RNeasy Mini Kit (Qiagen, Tokyo, Japan) according to the manufacturer’s protocol. The cDNA was then synthesized from total RNA using the High-Capacity cDNA Reverse Transcription Kit (Applied Biosystems Japan Ltd., Tokyo, Japan). A quantitative real-time PCR assay of individual cDNA was conducted with an ABI Prism 7500 instrument (Applied Biosystems Japan Ltd.) using TaqMan Gene Expression Assays (Applied Biosystems Japan Ltd.): TNF-α, Mm00443258-m1; IL-1β, Mm00434228_m1; IL-6, Mm00446190-mL; COX-2 (Ptgs2), Mm00478374-mL; iNOS (Nos2), Mm00440485-mL; and β-actin: Mm00607939-sl. The PCR cycling conditions were 50 °C for 2 min and 95 °C for 10 min, followed by 40 cycles of 95 °C for 15 s and 60 °C for 1 min. The expression level of each gene was normalized to the β-actin expression level. The 2 △△CT method with normalization to β-actin and group 5 was used. Each assay was performed in triplicate, and the average was calculated. 

### 2.5. Statistical Analysis

Measurements of the multiplicity of colonic lesions and the scores of the histological and immunohistochemical analyses were statistically analyzed using the Tukey or Bonferroni multiple comparison posttest. The incidences of colonic lesions between the groups were compared by Fisher’s exact probability test. The statistical analysis of differences in the mRNA expression was performed using the Kruskal-Wallis one-way ANOVA test. Differences were considered to be statistically significant at *p* < 0*.*05.

## 3. Results

### 3.1. Experiment 1 (the Eighteen-Week Experiment)

#### 3.1.1. General Observations in Experiment 1 (18-Week Study)

The three different Hrh antagonists-containing diets (500 ppm or 10 ppm) did not lead to any observable clinical toxicity. Histopathological examinations of the liver, kidneys, lungs, heart and spleens of the mice confirmed the findings (data not shown). The mean body weight and the mean weights of the three organs (liver, kidney, lung, heart and spleen) at week 18 did not significantly differ among the groups. The mean colon lengths of group 2 (AOM + DSS + 500 ppm terfenadine, 14.0 ± 0.3 cm, *p* < 0.001), 3 (AOM + DSS + 500 ppm cimetidine, 13.6 ± 0.4 cm, *p* < 0.05) and 4 (AOM + DSS + 10 ppm clobenpropit, 14.1 ± 0.5 cm, *p* < 0.001) were significantly longer than that of group 1 (AOM + DSS, 13.2 ± 0.5 cm). 

#### 3.1.2. Incidences and Multiplicities of Colorectal Mucosal Ulcers, High-Grade Dysplasia, Adenoma and Adenocarcinoma

**Figure 1:**
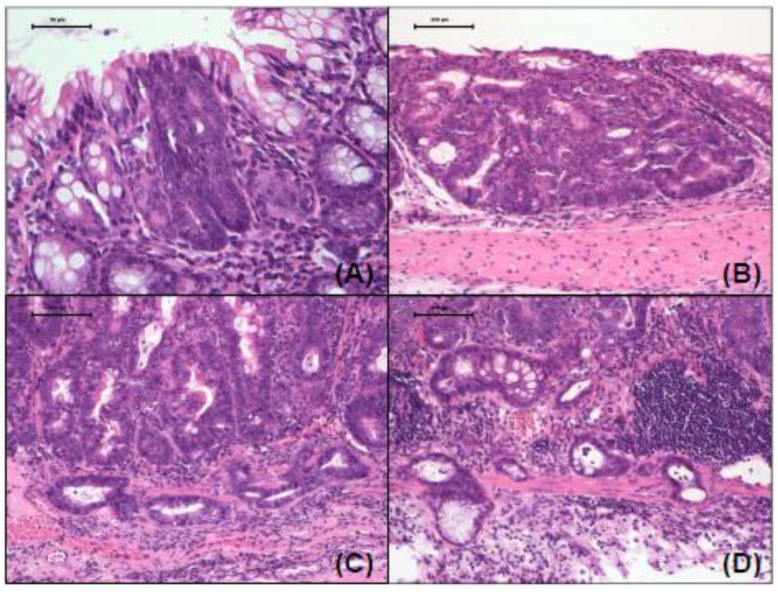
Representative images of proliferation colorectal lesions induced by AOM and DSS. (**A**) high grade dysplasia; (**B**) tubular adenoma; (**C**) adenocarcinoma without invasion; and (**D**) adenocarcinoma with invasion. H & E stain, 400×.

AOM/DSS treatment together with or without the antagonists resulted in the development of various colorectal lesions (Figure 1), while the treatment with antagonist alone and no treatment did not produce any colonic lesions (data not shown). Proliferative colonic lesions induced by AOM and DSS included high-grade dysplasia (Figure 1A), tubular adenoma (Figure 1B) and tubular adenocarcinoma without infiltration (Figure 1C) and with invasion (Figure 1D). The incidences and multiplicities of these lesions at week 18 are presented in Table 1. The incidences of the lesions in groups 2 through 4 were lower compared to those of group 1, although the incidences of adenocarcinoma and combined tumors (adenoma and adenocarcinoma) in group 2 were greater than those of group 1. The differences among the groups were no significantly different. On the other hand, the multiplicities of the lesions in groups 3 and 4 were significantly smaller than those of group 1 (*p* < 0.01 or *p* < 0.001).

#### 3.1.3. Biomarkers of Oxidative Stress and Antioxidant Defense Status

Results for the d-ROM and BAP tests are presented in Table 2. The d-ROM and BAP values and the d-ROM/BAP ratio obtained from the two tests did not significantly differ among the groups.

#### 3.1.4. Immunohistochemical Analysis of MCM2 (Cell Proliferation) and Caspase-3 (Apoptosis) in Colorectal Adenocarcinomas

**Figure 2:**
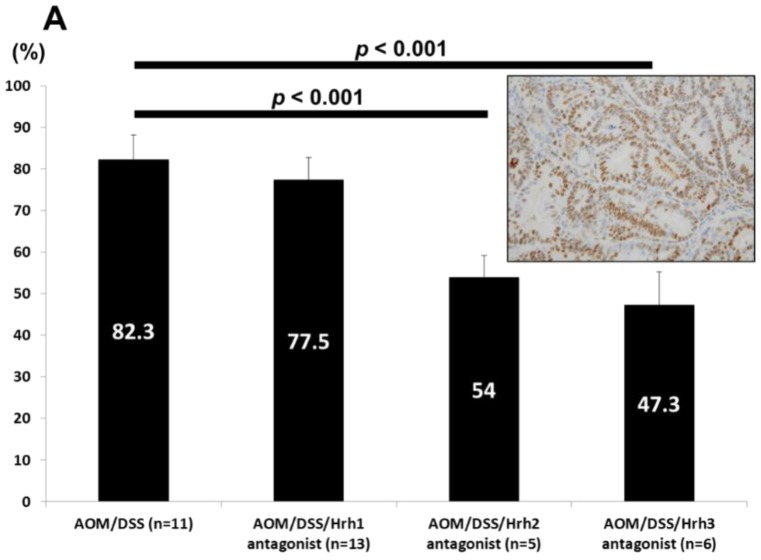

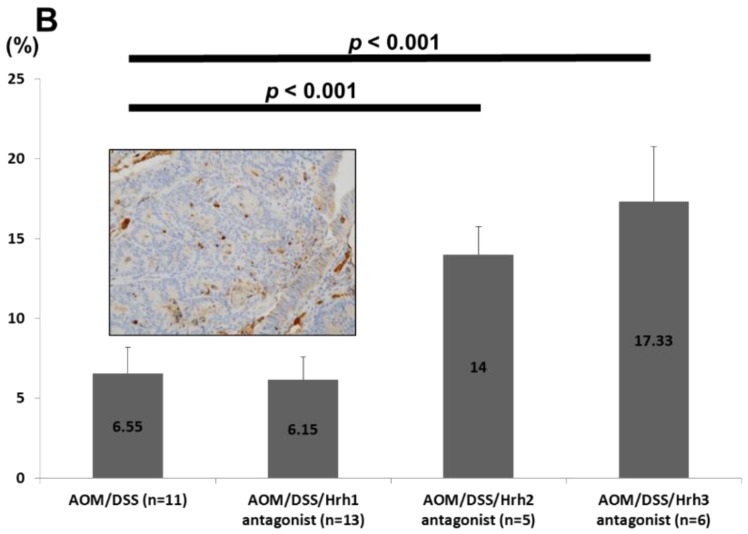
(**A**) Proliferating activities estimated by the MCM2-positive rate of cancer cells in Groups 1 through 4. Insert: MCM2 immunohistochemistry of adenocarcinoma developed in a mouse belonging to Group 1; (**B**) Apoptosis estimated by the caspase 3-positive rate of cancer cells in Groups 1 through 4. Insert: Caspase 3 immunohistochemistry of adenocarcinoma developed in a mouse belonging to Group 1.

As shown in Figure 2A, the mean MCM2-positive indices of colonic adenocarcinomas in groups 3 (*p* < 0.001) and 4 (*p* < 0.001) were significantly lower than that of group 1, indicating that dietary administration with Hrh2 and Hrh3 antagonists decreased cancer cell proliferation. However, the Hrh1 antagonist did not affect the proliferation activity of cancer cells. In contrast, cleaved caspase-3 positivity (Figure 2B) in adenocarcinomas that developed in groups 3 (*p* < 0.001) and 4 (*p* < 0.001) was significantly higher than that of group 1, suggesting the induction of apoptosis by dietary Hrh2 and Hrh3 antagonists in the cancer cells. The Hrh1 antagonist failed to induce apoptosis of cancer cells.

#### 3.1.5. Immunohistochemical Expression of Hrh1, Hrh2, Hrh3 and Hrh4 in Adenocarcinomas

**Figure 3:**
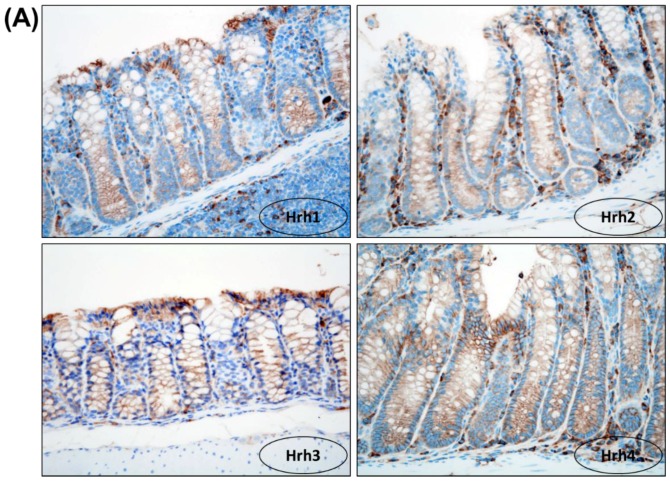

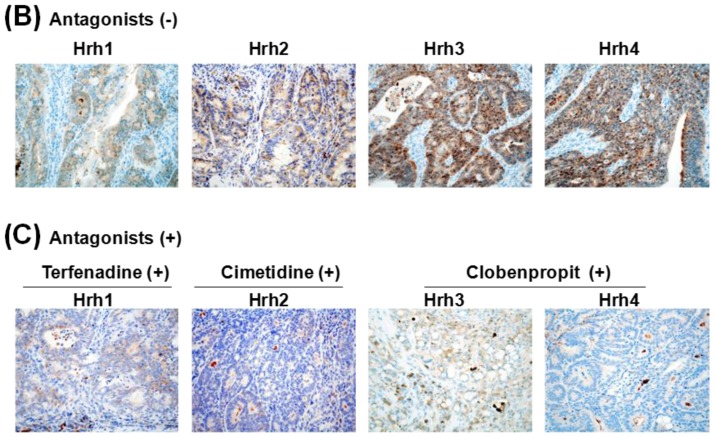
(**A**) Immunohistochemical expression of Hrh1, Hrh2, Hrh3 and Hrh4 in normal crypts. H & E stain, 400×; (**B**) Immunohistochemistry of Hrh1, Hrh2, Hrh3 and Hrh4 in adenocarcinomas developed in the mice of Group 1 (AOM + DSS) and (**C**) Groups 2 (AOM + DSS + terfenadine), 3 (AOM + DSS + cimetidine) and 4 (AOM + DSS + clobenpropit). Immunohistochemistry, 400×.

Hrh1, Hrh2, Hrh3 and Hrh4 positivity in the cell membrane and slightly in the cytoplasm of cryptal cells was observed (Figure 3A). As shown in Figure 3B, adenocarcinoma cells expressed Hrh1, Hrh2, Hrh3 and Hrh4. The order of intensity of the expression was Hrh3 = Hrh4 > Hrh2 > Hrh1. Dietary feeding of the three antagonists inhibited the expression of Hrh1, Hrh2, Hrh3 and Hrh4, respectively (Figure 3C). The inhibition of Hrh3 and Hrh4 was remarkable and treatment with clobenpropit lowered the expression of both Hrh3 and Hrh4.

### 3.2. Experiment 2 (the Four-Week Experiment)

#### 3.2.1. General Observations

The mean colon lengths of mice belonging to group 2 (DSS + 500 ppm terfenadine, 14.3 ± 1.3 cm, *p* < 0.05), 3 (DSS + 500 ppm cimetidine, 14.1 ± 1.4 cm, *p* < 0.05) and 4 (DSS + 10 ppm clobenpropit, 14.2 ± 1.8 cm, *p* < 0.05) were longer than that of group 1 (DSS alone, 11.8 ± 0.8 cm). The mean body and organ (liver, kidney and spleen) weights did not significantly differ among the groups. 

#### 3.2.2. Biomarkers of Oxidative Stress and Antioxidant Defense Status

DSS alone treatment significantly increased the values of the d-ROM test (144 ± 15 CARR U, *p* < 0.01) and the d-ROM/BAP ratio (0.066 ± 0.019, *p* < 0.05) compared to the untreated control (group 5, d-ROM test: 114 ± 13 CARR U and d-ROM/BAP ratio: 0.035 ± 0.004). Treatment with 500 ppm terfenadine (group 2), 500 ppm cimetidine (group 3) and 10 ppm clobenpropit (group 4) significantly lowered the values of d-ROM (group 2, 116 ± 8 CARR U, *p* < 0.01; group 3, 119 ± 11 CARR U, *p* < 0.01; group 4, 121 ± 12 CARR U, *p* < 0.01) and the d-ROM/BAP ratio (group 2, 0.039 ± 0.012, *p* < 0.01; group 3, 0.035 ± 0.007, *p* < 0.01; group 4, 0.037 ± 0.009, *p* < 0.01). Cimetidine treatment significantly increased the BAP value (3656 ± 579 mml/L, *p* < 0.01) when compared to that of the DSS alone group (group 1, 2372 ± 635 mml/L).

#### 3.2.3. mRNA Expression Levels of Inducible Inflammatory Enzymes and Proinflammatory Cytokines

**Figure 4:**
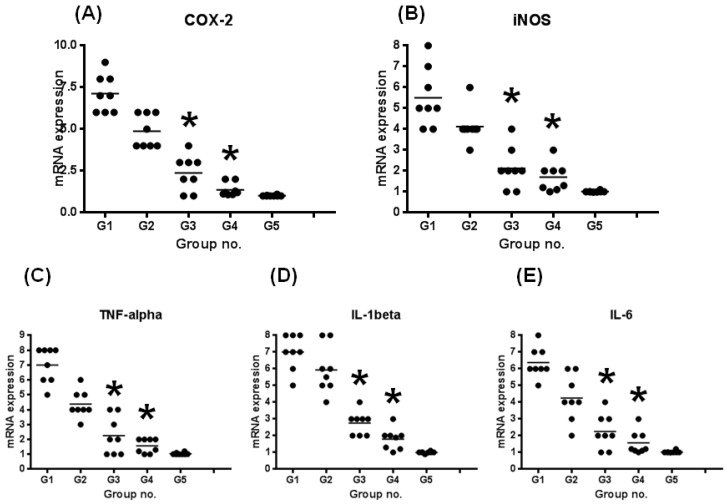
The mRNA expression levels of inducible inflammation-related enzymes and pro-inflammatory cytokines in colorectal tissue samples from mice treated with DSS with or without Hrh antagonists. Total RNA was extracted from the colorectal tissues of untreated and DSS/antagonists-treated mice. The mRNA levels of (**A**) COX-2, (**B**) iNOS, (**C**) TNF-α, (**D**) IL-1β and (**E**) IL-6 were measured by quantitative RT-PCR. Treatment with cimetidine or clobenpropit in the diet significantly decreased the mRNA expression of all molecules (* *p* < 0.05). The expression was normalized to the β-actin mRNA expression level. The data are expressed as the means ± SD from three independent assays (n=8 from each group). The ordinates show the relative mRNA expression (/β-actin).

Figure 4 shows the relative mRNA expression levels of COX-2 (Figure 4A), iNOS (Figure 4B), TNF-α (Figure 4C), IL-1β (Figure 4D) and IL-6 (Figure 4E), according to the RT-PCR analysis.

The expression levels of all genes in the mice treated with DSS alone (group 1) were increased in comparison with those in the untreated mice (group 5). Treatment with Hrh1, Hrh2 and Hrh3 antagonists decreased the mRNA expression of all molecules. Specifically, the mRNA expression of all mice treated with cimetidine (Hrh2 antagonist, group 3) or clobenpropit (Hrh3 antagonist, group 4) were significantly lower than group 1 (*p* < 0.05 for each comparison).

## 4. Discussion

In the current study, we observed the immunohistochemical expression of Hrh1, Hrh2, Hrh3 and Hrh4 in CRCs induced by AOM and DSS treatment. Although the Hrh1 antagonist (terfenadine) did not affect AOM-DSS-induced colorectal carcinogenesis in mice, the Hrh2 antagonist (cimetidine) and Hrh3 antagonist/inverse agonist (clobenpropit) significantly inhibited AOM-DSS-induced colorectal carcinogenesis in mice. These effects paralleled the findings obtained in the short-term experiment, in which the test compounds were able to improve oxidative stress caused by DSS and lowered the mRNA expression levels of inflammatory enzymes and cytokines in the inflamed colorectum of mice. The Hrh3 antagonist/inverse agonist, clobenpropit, is also known to be a partial Hrh4 receptor agonist. Therefore, our results suggest that Hrh2, Hrh3 and Hrh4 may be involved in inflammation-associated colorectal carcinogenesis in mice.

Toxicity of Hrh1-Hrh3 antagonists, especially terfenadine, is well-known and includes cardiotoxicities [39]. However, in the present study we did not observed the clinical toxicities during the experiment, Histopathological examination did not show abnormalities related to toxicity in major organs, including heart.

The mechanism(s) by which dietary cimetidine and clobenpropit significantly inhibit AOM/DSS-induced colorectal carcinogenesis include modulation of proliferation, apoptosis, the mRNA expression of two inflammatory enzymes (COX-2 and iNOS) and several cytokines (TNF-α, IL1β and IL-6), oxidative stress and the antioxidant status. Hrh1, Hrh2, Hrh3 and Hrh4 antagonists were previously reported to suppress the growth of human CRC in nude mice and chemically-induced colorectal carcinogenesis through suppression of cell proliferation, apoptosis induction and/or immune function [19,24,40]. The Hrh1 antagonist terfenadine was also found to induce apoptosis [30]. However, in the present study we did not observe apoptosis induction by terfenadine. We are the first to demonstrate that three antagonists of Hrh1, Hrh2 and Hrh3 affect oxidative stress and the antioxidant capacity, which were determined by the d-ROM and BAP tests, respectively. Both tests are highly reproducible and have been widely used to investigate oxidative stress and antioxidant states [35].

In the current study, the Hrh1 antagonist terfenadine did not affect AOM/DSS-induced murine colorectal carcinogenesis, which was similar to the previously reported finding that treatment with terfenadine or a selective Hrh2 antagonist ranitidine alone did not influence the growth of human hepatocellular cancer cell lines HuH-6 and HA22T/VGH [29]. Although terfenadine was reported to induce apoptosis and inhibit the growth of human prostate, liver and colon cancer cell lines [31,41] and a melanoma cell line [30], our results showed that terfenadine did not induce apoptosis in CRC cells. The reason for this discrepancy remains unknown, but may be due to differences in the methods of administration. In this study, terfenadine treatment did not influence the antioxidant status or oxidative stress, as determined by the BAP and d-ROM tests, respectively. In contrast to the effects of terfenadine, cimetidine and clobenpropit significantly inhibited inflammation-associated colorectal carcinogenesis. Although some Hrh2 antagonists, such as ranitidine and famotidine, were found to lack tumor-inhibitory effects [42], cimetidine was able to suppress the growth of CRC implants in mice [26], chemically-induced intestinal cancers in rats [43] and human CRC [23] by inhibition of angiogenesis [26]. In the current study, we did not determine the effects of cimetidine on tumor angiogenesis. However, dietary cimetidine significantly suppressed the expression of cytokines and inflammatory enzymes and oxidative stress in the inflamed colorectum. Interestingly, cimetidine was reported to lower the expression of cytokines in a xenograft colon cancer cell line (CT-26) in BALB/c mice [25]. Thus, our results are in accordance with a previous report that demonstrated cimetidine to be an anti-tumor agent [40]. As noted in the case of cimetidine, a Hrh3 antagonist/Hrh4 agonist (clobenpropit) was able to inhibit AOM/DSS-induced murine colorectal carcinogenesis by modulating oxidative stress, the antioxidant status and the mRNA expression of certain inflammatory enzymes and cytokines. Recently, Meng *et al*. have reported interesting findings suggesting that clobenpropit suppressed the progression of human cholangiocarcinoma cells through disruption of the epithelial-mesenchymal transition (EMT) and metastasis using in vitro and in vivo model systems [34]. In another interesting report, inhibition of the Hrh4 expression in CRC resulted in inhibition of tumor growth and tumor progression [32]. In addition, clobenpropit enhanced the anti-tumor effects of gemcitabine in pancreatic cancer through inhibition of the EMT process [44]. Taken together, these findings suggest new clues for the application of Hrh3 antagonists and Hrh4-specific agonist or antagonists in the therapy or prevention of CRC [19].

In addition to immunotherapy, pro-inflammatory cytokines secreted by innate and adaptive immune cells could be potential targets for IBD treatment [38]. Similarly, some cytokines, such as TNF-α [16], IL-1β [45] and NF-κB [38], are potential molecular targets for inflammation-associated CRC [8], as demonstrated in the present in study.

## 5. Conclusions

In conclusion, our findings showed that Hrhs are involved in colitis-related colorectal carcinogenesis in mice. Our results indicated that cimetidine (Hrh2 antagonist) and clobenpropit (Hrh3 antagonist/inverse agonist) may be potential chemopreventive agents against inflammation-associated colorectal carcinogenesis.

## Figures and Tables

**Table 1:** Incidences and multiplicities of colonic lesions in all groups (18-week experiment).

Group No.	Treatment	No. of Mice Examined	Mucosal Ulcer	High-Grade Dysplasia	Adenoma (AD)	Adeno-Carcinoma (ADC)	Total Tumor (AD+ADC)
**1**	AOM + DSS	20	10/20 (50%)	15/20 (75%)	11/20 (55%)	11/20 (55%)	13/20 (65%)
			1.87 ± 1.81 ^a^	3.93 ± 2.43	2.40 ± 2.35	2.73 ± 3.45	5.13 ± 4.93
**2**	AOM + DSS + 500 ppm terfenadine	20	10/20 (50%)	13/20 (65%)	10/20 (50%)	13/20 (65%)	14/20 (70%)
			1.14 ± 0.95	3.29 ± 2.37	1.50 ± 1.70	2.50 ± 1.34	4.00 ± 2.11
**3**	AOM + DSS + 500 ppm cimetidine	20	6/20 (30%)	11/20 (55%)	6/20 (30%)	5/20 (25%)	8/20 (40%)
			0.53 ± 0.74 ^b^	1.13 ± 1.13 ^c^	0.53 ± 0.74 ^b^	0.60 ± 1.12 ^b^	1.13 ± 1.73 ^c^
**4**	AOM + DSS + 10 ppm clobenpropit	20	8/20 (40%)	13/20 (65%)	7/20 (35%)	6/20 (30%)	8/20 (40%)
			0.60 ± 0.63 ^b^	1.67 ± 1.29 ^b^	0.73 ± 0.96 ^b^	0.60 ± 0.91 ^b^	1.33 ± 1.72 ^c^
**5**	500 ppm terfenadine	8	0/8 (0%)	0/8 (0%)	0/8 (0%)	0/8 (0%)	0/8 (0%)
**6**	500 ppm cimetidine	8	0/8 (0%)	0/8 (0%)	0/8 (0%)	0/8 (0%)	0/8 (0%)
**7**	10 ppm clobenpropit	8	0/8 (0%)	0/8 (0%)	0/8 (0%)	0/8 (0%)	0/8 (0%)
**8**	None	8	0/8 (0%)	0/8 (0%)	0/8 (0%)	0/8 (0%)	0/8 (0%)

^a^ Mean ± SD; ^b-d^ Significantly different from group 1 by one-way ANOVA followed by Tukey-Kramer test (^b^ *p* < 0.01 and ^c^ *p* < 0.001).

**Table 2:** Measurement of oxidative stress markers (18-week experiment).

Group No.	Treatment	No. of Mice Examined	d-ROM ^a^ (CARR U ^b^)	BAP ^a^ (mM/L)	d-ROM/BAP Ratio
1	AOM + DSS	20	126 ± 11 ^c^	2931 ± 302	0.04361 ± 0.00823
2	AOM + DSS + 500 ppm terfenadine	20	133 ± 11	2958 ± 470	0.04795 ± 0.00591
3	AOM + DSS + 500 ppm cimetidine	20	123 ± 26	1630 ± 987	0.09594 ± 0.05643
4	AOM + DSS + 10 ppm clobenpropit	20	131 ± 15	2328 ± 1088	0.07353 ± 0.02484
5	500 ppm terfenadine	8	138 ± 9	2658 ± 909	0.04968± 0.01197
6	500 ppm cimetidine	8	130 ± 8	2753 ± 1802	0.09091 ± 0.08894
7	10 ppm clobenpropit	8	147 ± 10	1405 ± 801	0.15870 ± 0.06784
8	None	8	142 ± 18	2477 ± 954	0.08182 ± 0.05208

^a^ d-ROM = derivatives of reactive oxygen metabolites, and BAP = biological antioxidant potential; ^b^ 1 Carr U is equivalent to 0.08 mg H_2_O_2_/dL; ^c^ Mean ± SD.
