# Erratum: Tanaka, T. et al. Cimetidine and Clobenpropit Attenuate Inflammation-Associated Colorectal Carcinogenesis in Male ICR Mice. *Cancers*, 2016, *8*, 25

**DOI:** 10.3390/cancers9070080

**Published:** 2017-07-05

**Authors:** Takuji Tanaka, Takahiro Kochi, Yohei Shirakami, Takayuki Mori, Ayumi Niwa, Naoki Watanabe, Hisataka Moriwaki, Masahito Shimizu

**Affiliations:** 1 Department of Diagnostic Pathology (DDP) and Research Center of Diagnostic Pathology (RC-DiP), Gifu Municipal Hospital, 7-1 Kashima-cho, Gifu City, Gifu 500-8513, Japan; mulmirry@yahoo.co.jp (A.N.); naoki@watanabe.name (N.W.); 2 Department of Tumor Pathology, Gifu University Graduate School of Medicine, 1-1 Yanagido, Gifu City, Gifu 501-1194, Japan; 3 Department of Gastroenterology/Internal Medicine, Gifu University Graduate School of Medicine, 1-1 Yanagido, Gifu City, Gifu 501-1194, Japan; kottii924@yahoo.co.jp (T.K.); shirakamiyy@yahoo.co.jp (Y.S.); hmori@gifu-u.ac.jp (H.M.); shimim-gif@umin.ac.jp (M.S.); 4 Department of Pharmacy, Ogaki Municipal Hospital, 4-86 Minaminokawa-cho, Ogaki 503-8502, Japan; ta_mori04@yahoo.co.jp

The authors wish to make the following correction to their paper [1]. The author Ayumi Kurata’s name should be changed to Ayumi Niwa.

The authors would like to apologize for any inconvenience caused. The change does not affect the scientific results. The manuscript will be updated and the original will remain online on the article webpage. 
